# Positive epigenetic regulation loop between AR and NSUN2 promotes prostate cancer progression

**DOI:** 10.1002/ctm2.1028

**Published:** 2022-09-28

**Authors:** Wenkai Zhu, Fangning Wan, Wenhao Xu, Zheng Liu, Junjie Wang, Hena Zhang, Shenglin Huang, Dingwei Ye

**Affiliations:** ^1^ Department of Urology Fudan University Shanghai Cancer Center Shanghai China; ^2^ Qingdao Institute School of Life Medicine Department of Urology Fudan University Shanghai Cancer Center, Fudan University Qingdao China; ^3^ Fudan University Shanghai Cancer Center, and the Shanghai Key Laboratory of Medical Epigenetics, Institutes of Biomedical Sciences Fudan University Shanghai China

**Keywords:** androgen receptor, AR‐V7, cluster, castration‐resistant prostate cancer, cytosine‐5 methylation, NSUN2, prostate cancer

## Abstract

**Background:**

Prostate cancer (PCa) is a major type of cancer in man worldwide. Androgen deprivation therapy (ADT) and the next‐generation androgen receptor (AR) pathway inhibitors have acquired great success in treating PCa. However, patients treated with ADT or AR targeted therapy are inevitably developing into castration‐resistant prostate cancer (CRPC) or becoming drug resistance. The role of mRNA 5‐methylcytosine (m5C) modification in cancers is largely unknown. This study aimed to explore the role of the m5C methyltransferase NSUN2 in Prostate cancer (PCa).

**Methods:**

The expression of NSUN2 and its clinicopathological impact were evaluated in PCa cohorts. The effect of NSUN2 on the biological characteristics of PCa cells was investigated on the basis of gain‐offunction and loss‐of‐function analyses. Subcutaneous models further uncovered the role of NSUN2 in tumor growth. Epi‐transcriptome assays with RNA bisulfite sequencing (RNA‐BisSeq) analysis and in vitro enzyme reaction assays were performed to validate the targeted effect of NSUN2 on AR. AR‐binding sites in the NSUN2 promoter were investigated by ChIP and luciferase assays to uncover the interplay between NSUN2 and AR signaling. RIP‐qPCR and EMSA methods were performed to confirm that YBX1 binds to AR m^5^C sites.

**Results:**

NSUN2 is highly expressed in PCa and predicts poor outcome. NSUN2 plays roles as a PCa oncogene both in vitro and in vivo. Depletion of NSUN2 results in decreased expression and activities of AR, including AR‐V7. Mechanistically, NSUN2 posttranscriptionally stabilized AR by cluster m^5^C modification in a m5CYBX1‐dependent manner. Strikingly, treatment with enzalutamide, an effective AR inhibitor, reduces NSUN2 expression and decreases the m5C modification level in prostate cancer cells. Finally, we found that AR transcriptionally regulates NSUN2.

**Conclusion:**

NSUN2 stabilizes AR mRNA through cluster 5‐methylcytosine modification and activates a positive feedback loop to promote prostate cancer.

## Graphical abstract

- NSUN2 expression is upregulated in PCa and associated with a worse prognosis.
- AR mRNA is modified by NSUN2 and is stabilized in an m5C‐YBX1‐dependent manner.
- The m5C modification located in the 5′ region of AR mRNA, influenced several AR variants including AR‐V7.
- NSUN2 expression is also transcriptionally regulated by AR and can respond to ADT and ARSI treatment.

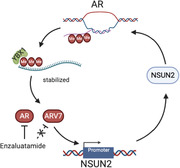

## 1 INTRODUCTION

Prostate cancer (PCa) is the most common male malignancy in the West, and its incidence is rapidly increasing in Asia.^1^, ^2^ The most important therapeutic target in PCa is the androgen receptor (AR).^3^ Androgen deprivation therapy (ADT) has long been the cornerstone of PCa treatment, especially metastatic PCa treatment.^4^, ^5^ However, metastatic PCa treated with ADT inevitably develops castration‐resistant prostate cancer (CRPC).^6^, ^7^ Since 2012, the FDA has approved several new AR inhibitors, such as abiraterone, enzalutamide, and apalutamide, to treat CRPC.^5^, ^8^, ^9^, ^10^ These drugs, prolonged the overall survival of PCa patients. However, AR variants such as AR splice variant 7 (AR‐V7) and AR‐V9 can stimulate AR inhibitor resistance, as they can induce self‐activation without androgen binding.^11^, ^12^ Therefore, it is really urgent to uncover the resistance mechanisms and develop novel therapeutics.

The mechanisms behind CRPC can be complex. It occurs primarily through AR signalling reactivation in a ligand‐dependent or ligand‐independent manner.^13^ Resistance can occur for many reasons. It can be intrinsic, such as a mutation of *TP53*, or acquired, such as *AR* amplification or mutations.^14^ *AR* amplification or mutations are the most common recurrent somatic gene alterations in mCRPC, accounting for approximately 62.7% in a biopsy study.^15^ For these reasons, AR is still an important therapeutic target in CRPC treatment.

RNA 5‐Methylcytosine (m^5^C) modifications were first discovered in abundant and stable tRNAs and rRNAs.^16^ Recently, the mRNA m^5^C modification has been identified to regulate mRNA metabolism and translocation.^17^, ^18^ It has been shown that mRNA m^5^C modification could impact various biological events, such as embryonic development, myelopoiesis^19^ and tumorigenesis.^17^ There are nine subfamilies of cytosine‐5 methyltransferases (RCMTs). However, only three subfamilies (RCMT2, RCMT7 and RCMT8) are found in eukaryotic species. NSUN2 and NSUN6 are the two known m^5^C methyltransferases (‘writers’) of mRNA to date.^18^, ^20^, ^21^ YBX1 and ALYREF, discovered by Yang, are the only known m^5^C 'readers.'^17^, ^18^ NSUN2 has been reported to be involved in promoting the progression of some tumours. It can modify the mRNA of HDGF to promote the progression of bladder cancer.^17^ In gastric cancer, NSUN2 represses p57^Kip2^ in an m^5^C‐dependent manner.^22^ NSUN2 is important for cervical cancer progression.^23^ Autotaxin mRNA can be methylated by NSUN2 and promote tumour migration and invasion.^24^ As a new epigenetic regulation mechanism in tumour biology, whether or how NSUN2 plays roles in PCa, even in CRPC and drug resistance remains largely unknown. YBX1 acts as a reader to promote many kinds of cancers that have been reported. YBX1 can form a positive feedback loop with lncRNA to activate the FOXA1 transcription network.^25^ FOXA1 can interact with AR and help shape the AR signalling.^26^ These indicate that NSUN2 and YBX1 may act in an axis to influence the AR signalling. The aberrant AR signalling contributes to PCa progression and even its formation.

## 2 MATERIALS AND METHODS

### 2.1 Cell lines, antibodies, chemical inhibitors

The human PCa cell lines C4‐2, LNCaP, and 22RV1 were used. Details of the antibodies are in Supporting Information. Actinomycin D was purchased from Abcam, Shanghai. Enzalutamide (MDV3100) was purchased from Selleck, Shanghai.

### 2.2 Cell culture, siRNA and plasmid transfection, lentivirus generation and infection

C4‐2, LNCaP, 22RV1 were maintained in RPMI 1640 basic medium (Gibco, C11875500BT) with 10% FBS (Gibco, A3160802).

NSUN2 siRNAs were synthesized by Biosun Company.

Lipofectamine 3000 (Invitrogen) was used for plasmid and siRNA transfection following the manufacturer's protocols.

### 2.3 RNA m^5^C quantification by LC/MS/MS

mRNA from C4‐2 cells was purified using the Dynabeads™ mRNA Purification Kit. The purified mRNA is digested into single nucleotides and quantitatively detected by LC/MS/MS by Shanghai Biotree Co., Ltd. The content of m^5^C and cytosine was calibrated by the standard curve generated by the standards (purchased from APExBIO). Following the standard protocol, add 200‐300 ng of mRNA to 25 µl of dilution buffer, digest with nuclease P1 (1 U) for 2 h at 42°C, followed by alkaline phosphatase (1 U) and NH4HCO3 (1 M, 3 µl) treated at 37°C incubate for 2 h, and finally filter through a filter for mass spectrometry analysis.

### 2.4 Dot blot assay

Samples were spotted onto Amersham HybondTM‐N+ membranes (GE Healthcare) and cross‐linked with UV, then washed with wash buffer for 7 min and blocked for 1 h at room temperature, with anti‐m^5^C antibody (Abcam, ab214727, 1:1000) at 4°C. Then incubated with secondary antibody (Proteintech, SA00001‐2) for 1 h and exposed with LAS 4000 mini for 30 seconds.

### 2.5 Immunocytochemical staining and cell imaging

Cells were fixed with methanol for 15 min and then permeabilized with 0.1% Triton X‐100 on ice for 15 min. This was followed by blocking with 10% FBS for 1 h, followed by incubation with primary antibody overnight at 4°C, followed by incubation with secondary antibody for 1 h, and finally mounting with DAPI‐containing mounting medium (P36931, Invitrogen). The fluorescence intensity was quantified by ImageJ.

### 2.6 Secreted PSA quantification

Cells were pre‐treated for 72 h. Then, add 50 µl of supernatant to the chip (a rapid quantitative immunoassay analyser) (FREND™ System, NanoEnTek Inc. Korea) and wait for 5 min. Finally, the chip was put into the detection instrument (FREND™ System, NanoEnTek Inc. Korea).

### 2.7 RNA probe synthesis

The sequence probes were designed in our laboratory. These probes were synthesized by a standard RNA synthesis kit (NEB, E2050) following the manufacturer's protocols.

### 2.8 RNA pull‐down assay

Cell lysates were prepared with RIPA cell lysis buffer. Briefly, mix 50 µl streptavidin magnetic beads with 50 pmol 3′‐biotin‐labelled RNA probes and incubated for 30 min with agitation. After incubation, add 100 mg protein lysate and incubated for 60 min at 4°C with agitation. The probe sequences were as follows:

probe‐mut: AugAugAugAagaagAagAagAagAagaagaagaagaagaagaagaagaagaagaagaagaagaagaagaag

probe‐normal: CugCugCugCagcagCagCagCagCagcagcagcagcagcagcagcagcagcagcagcagcagcagcagcag

### 2.9 Biochemistry assay for RNA m^5^C transferase reaction in vitro

0.15 nmol probe and 0.15 nmol NSUN2 protein were added into a buffer contain 0.8 mM SAM, 1.5 mM MgCl_2_, 80 mM KCl, 0.2 U µl^−1^ RNasin, 0.2 U µl^−1^ DNasin, 4% glycerol, 10 mM DTT and 15 mM HEPES (pH 7.9) incubated at 16°C overnight.

### 2.10 Bisulphite conversion

Purified mRNA (500 ng) was converted with the EZ RNA Methylation Kit (Zymo Research).

### 2.11 RNA‐seq

#### 2.11.1 Library preparation and deep sequencing

We collected samples from Fudan University Shanghai Cancer Center (FUSCC). The tissues were put into a homogenization tube, grinding beads and 1 ml of TRIzol (Invitrogen) were added. After grinding, it was centrifuged at 12 000 rpm/min for 15 min, and the supernatant was collected. RNA was purified by TRIzol (Invitrogen). All RNA libraries were constructed with RNA Library Prep Kit for Illumina (E7760), and ribosomal RNA (rRNA) was deleted with the NEBNext rRNA Depletion Kit (E7405). Then, we sent the libraries to a sequencing facility for Illumina 6G sequencing. The raw read qualities were evaluated by FastQC.

#### 2.11.2 RNA‐Seq data processing

Raw reads were filtered using Trimmomatic to remove low‐quality bases and adaptor sequences and then aligned to the GRCh38 human genome with GENCODE v29 gene annotation using STAR. Gene expression levels were calculated with FPKM values by normalizing gene counts from feature counts. All statistical analyses were performed in R.

### 2.12 PCR and Sanger sequencing

Five hundred nanograms of in vitro m^5^C transferase reaction probes, nonreaction probes and negative control R‐Luc were converted (see above). Then, the target sequences of the m^5^C transferase reaction probes, nonreaction probes and R‐Luc were amplified by PCR and subjected to Sanger sequencing.

### 2.13 RIP

The EZ‐Magna RIP kit (NO.17‐701) was used. Briefly, cells were lysed on ice with RIP lysis buffer. Then, 50 µl protein A/G magnetic beads, 100 µl cell lysis and 900 µl RIP buffer were added to a clean microtube and rotated at 4°C for 3 h. Ten microliters of cell lysate was kept as input and stored at −80°C. Finally, the RNA was purified, collected and converted to cDNA for qPCR.

### 2.14 ChIP

The ChIP assay was performed with EZ‐Magna ChIP G (Millipore, 17–409). Briefly, the cell lysates were prepared with cell lysis buffer and nuclear lysis buffer with protease inhibitor cocktail II. Then, the cell lysates were ultrasonicated to break the genomic DNA into 200 ‐1000 bp pieces. After that, the treated cell lysates, antibodies and protein G magnetic beads were mixed and incubated at 4°C with rotation. Finally, the DNA fragments were purified and collected for qPCR and sequencing.

### 2.15 mRNA stability assay

Each sample was harvested at 0, 3, 6, and 7 h after treatment with actinomycin D (2 µM). Total RNA was isolated and converted to cDNA for RT‐qPCR analysis.

### 2.16 Luciferase reporter assay

The modification site sequence of AR was inserted downstream of the promoter of the pGL3‐promoter luciferase vector (Vigenebio, Maryland, USA). C4‐2 cells were co‐transfected with a mixture of 2000 ng pGL3‐promoter‐AR‐Luciferase reporter vector and 1000 ng pRL‐TK vector. The relative luciferase activity was measured with a dual luciferase reporter assay system (Promega, Madison, WI).

### 2.17 EMSA

Biotin‐labelled RNA oligonucleotides were produced according to the above protocol. Recombinant human YBX1 proteins were purchased from Abcam (ab187443). The YBX1 proteins were mixed with probes in a binding buffer for 30 min at room temperature. The product was separated with 6% native PAGE gel and transferred to a nylon membrane. Membranes were then UV cross‐linked, blocked for 15 min, and incubated with HRP‐linked streptavidin (1:300) for 15 min. Add chemiluminescent substrate and incubate for 1‐3 min to expose with LAS 4 000 mini for 1‐5 min. The probe sequences were as follows: 5′‐cuuuccagaaucug……cugcugcugcagcagcagcagcagcagcagcagcagcagcagcagcagcagcagcagcagcagcagcagcagcagcaagagacuagccccaggcagcagcagcagcagcagggugaggaugg……gccagcaaggggcugcc‐3′.

### 2.18 In vivo xenograft assay

This study was approved by Fudan University (2020 FUSCC JS‐243). All treatments were administered according to the guidelines of the Institutional Animal Care and Use Committee.

A total of 10^7^ C4‐2 (sh‐NSUN2‐2, OE‐NSUN2 or control) cells were mixed with Matrigel and injected into the backside of 6‐week‐old male BALB/cA nude mice. The animals were checked every 5 days. The tumours were weighed after the mice were sacrificed.

### 2.19 Patients collection

A total of 12 452 cancer patients with available level 3 RNA‐seq data were recruited from the TCGA database in the website: http://xena.ucsc.edu. Clinical data and gene expression profiles of 497 PCa tissues and 52 normal tissues were obtained. Besides, 88 high‐risk PCa patients enrolled from FUSCC, with pathology and electronic medical records provided. Samples of PCa and normal prostate tissues were collected during surgery or biopsy, then processed and stored at the FUSCC tissue bank.

### 2.20 Immunoblotting

Primary antibodies against NSUN2, AR, AR‐V7, YBX1 and β‐actin were used for the immunoblot assay in this study. Detailed information is listed in Supporting Information.

### 2.21 Statistical analysis

Statistical analysis was performed with SPSS 22.0 software (SPSS, Chicago, Illinois) and R. Continuous variables were compared with a *t*‐test, while categorical variables were compared with a χ2 test. All significance tests were two‐sided, and differences with a *p‐*value < .05 were considered significant. The cut‐off value was defined via median value or using the 'survminer' R package.

## 3 RESULTS

### 3.1 NSUN2 upregulation in PCa associated with worse prognosis

**FIGURE 1:**
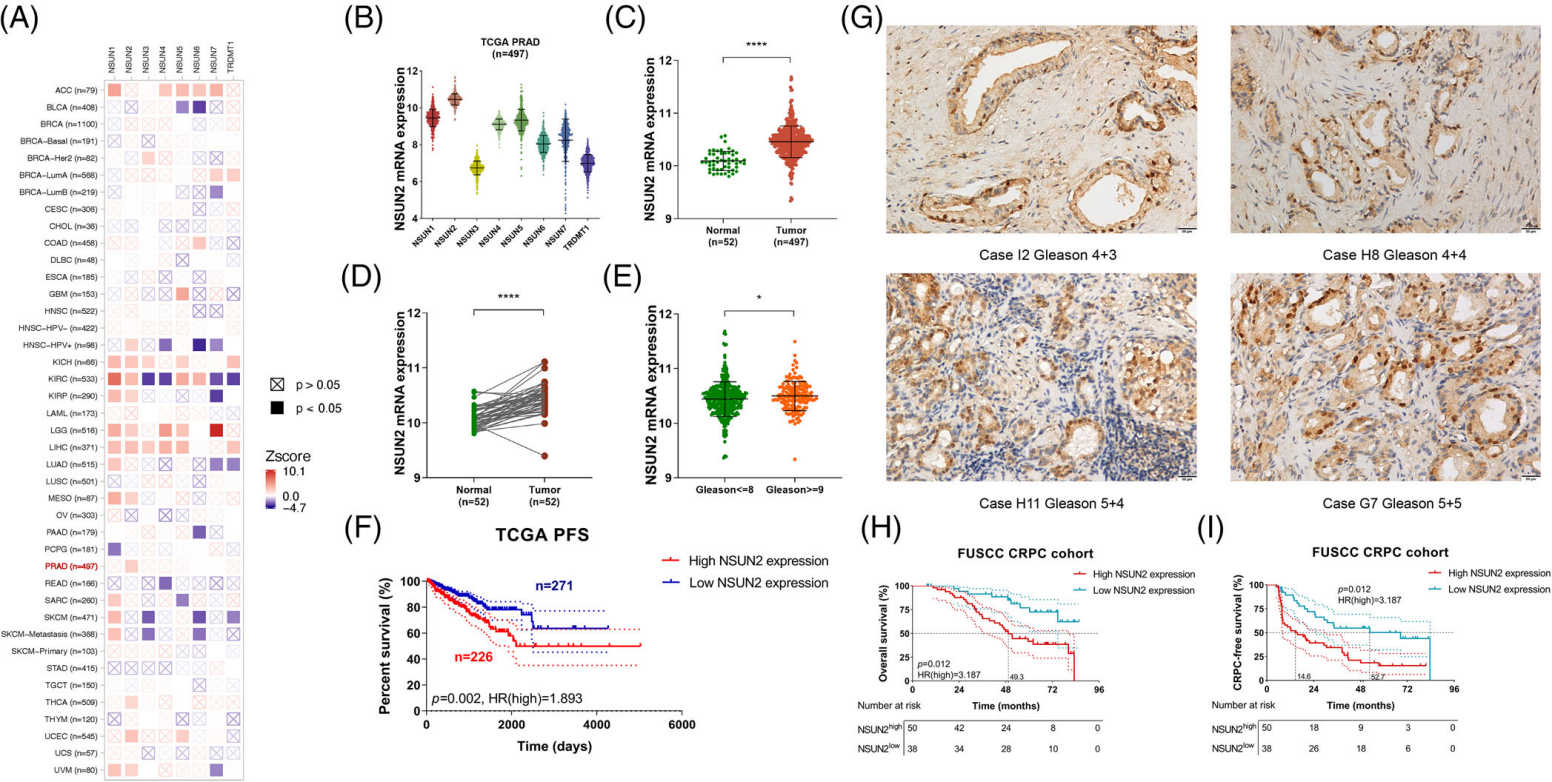
NSUN2 upregulation in PCa indicates worse prognosis. (A) Pan‐cancer overall survival outcome analysis of cytosine‐5‐methyltransferases in TCGA cohorts using Cox proportional hazard model. (B) Box and whisker dot plot of cytosine‐5‐methyltransferases expression levels in PCa tissues from the TCGA cohort. (C) Scatter plot of NSUN2 mRNA expression in normal tissues and PCa tissues from the TCGA cohort. (D) Scatter diagram of NSUN2 mRNA expression in 52 paired PCa and adjacent normal tissues. (E) Scatter plot of NSUN2 mRNA expression in tissues from patients with high Gleason scores (Gleason > = 9). (F) K‐M curves of the PFS of 497 patients from the TCGA cohort. (G) Representative images (×40 magnification) of NSUN2 expression in prostate cancer tissues examined by IHC. (H) Kaplan–Meier analysis shows that high expression of NSUN2 predicts poor OS for PCa patients. The bottom panel shows the number of patients at risk of the corresponding node. (FUSCC cohort; *n* = 88 cases). (I) Kaplan–Meier analysis shows that high expression of NSUN2 predicts poor CRPC‐free survival for PCa patients. The bottom panel shows the number of patients at risk of the corresponding node. (FUSCC cohort; *n* = 88 cases). PCa, prostate cancer

NOP2/Sun RNA methyltransferase gene families and TRDMT1 are known human cytosine‐5‐methyltransferases responsible for the methylation of RNA carbon 5 in cytidine (m^5^C, 5‐methylcytidine).^27^, ^28^, ^29^ In the Cancer Genome Atlas (TCGA) pan‐cancer result, NSUN2 was the only poor outcome predictor of overall survival (OS) in PCa (Figure 1A). Also, in the TCGA prostate adenocarcinoma (PRAD) cohort, NSUN2 was the most abundant gene among the cytosine‐5‐methyltransferases in PCa (Figure 1B). The clinicopathological parameters were shown in Table S1. *NSUN2* mRNA expression levels were significantly higher in 497 tumour samples than in 52 normal tissues (*p* < .01, Figure 1C). In 52 paired PCa and adjacent normal tissues, *NSUN2* mRNA expression levels were markedly elevated in tumour tissues (*p* < .01, Figure 1D). *NSUN2* mRNA expression significantly increased in patients with high Gleason scores (*p* < .05, Gleason > = 8, Figure 1E). High NSUN2 expression was associated with shortened PFS in 497 TCGA PCa patients (*p* = .002, HR = 1.893, Figure 1F) and shortened OS in 492 TCGA PCa patients (*p* = .048, HR = 4.2, Figure S1A). In contrast, NSUN6 showed no outcome predictive values in either OS (*p* = .63, HR = 0.74, Figure S1B) or DFS (*p* = .47, HR = 1.2, Figure S1C) in the same cohort. We performed immunohistochemistry (IHC) staining of a cohort of 88 high‐risk PCa patients obtained from the Fudan University Shanghai Cancer Center (FUSCC). The clinicopathological parameters were shown in Table S2. Representative images were shown in Figure 1G. High NSUN2 expression was associated with poor OS (*p* = .012, HR = 3.187, Figure 1H) and shorter CRPC‐free survival (*p* < .001, HR = 2.467, Figure 1I). These results demonstrated that the upregulation of NSUN2 is a frequent oncogenic event in human PCa patients.

### 3.2 NSUN2 regulates the proliferation, invasion and migration of PCa cells in vitro and in vivo

**FIGURE 2:**
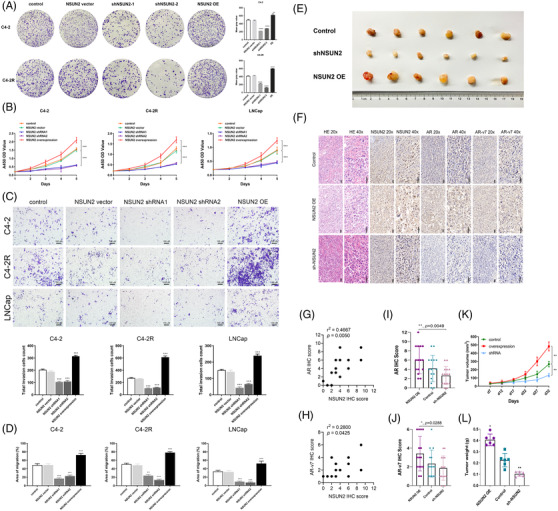
NSUN2 regulates the proliferation, invasion and migration of PCa cells both in vitro and in vivo. (A) Colony formation assay of C4‐2 and C4‐2R cells with NSUN2 knockdown or OE (*n* = 3 independent experiments). The *p* values were determined using a two‐sided unpaired student's *t*‐test. (B) CCK‐8 assay of C4‐2, C4‐2R and LNCaP cells with NSUN2 knockdown or OE (*n* = 3 independent experiments). The *p* values were determined using a two‐sided unpaired student's *t*‐test. (C) Transwell assay of C4‐2, C4‐2R and LNCaP cells with NSUN2 knockdown or OE (*n* = 3 independent experiments). The *p* values were determined using a two‐sided unpaired student's *t*‐test. (D) Wound healing assay of C4‐2, C4‐2R and LNCaP cells with NSUN2 knockdown or OE (*n* = 3 independent experiments). The *p* values were determined using a two‐sided unpaired student's *t*‐test. (E) A xenograft mouse model was constructed by subcutaneously injecting 6‐week‐old BALB/cA nude mice with C4‐2 cells. Decreased tumour volume and weight were observed in the NSUN2 shRNA‐2 group, and increased tumour size was observed in the NSUN2 OE group compared with the control group. (F) HE staining and IHC were implemented to detect the expression levels of NSUN2, AR and AR‐V7 using tumour tissues harvested from xenograft model mice. IHC of tumour tissues from the negative control group showing (G) NSUN2, AR and (H) AR‐V7 expression. Plots showing the IHC scores of (I) AR and (J) AR‐V7. Plots of tumour (K) volume and (L) weight

To investigate whether NSUN2 could influence the progression of PCa and be a potential therapeutic target, a series of experiments were conducted. LNCaP (androgen‐sensitive PCa cell), C4‐2 (castration‐resistant PCa cell) and C4‐2R (enzalutamide resistant PCa cell)^7^ cells were transfected with shRNAs targeting *NSUN2* or lentiviral overexpression (OE) vectors. Colony formation assays revealed markedly fewer colonies when NSUN2 knocked down, consistently, more colonies when NSUN2 overexpression (Figure 2A). The CCK‐8 assay showed that, in the NSUN2 overexpression group, the cell proliferation ability was significantly increased, while in the NSUN2 knockdown group, it was significantly decreased. (Figure 2B). Transwell assays showed a significantly decreased number of invading C4‐2, C4‐2R or LNCaP cells in the shRNA groups and an increased number of invaded C4‐2, C4‐2R or LNCaP cells in the NSUN2 OE group compared with the negative control group (Figure 2C). The wound healing assay showed that, in the NSUN2 OE group, the cell migration ability was significantly improved, while in the NSUN2 knockdown group, it was significantly reduced (Figure 2D, Figure S2). In the NSUN2 OE group, the tumour volume was significantly increased, while in the NSUN2 knockdown group, the tumour volume was significantly decreased (Figure 2E). Haematoxylin‐eosin (HE) staining and IHC were implemented to detect the expression levels of NSUN2, AR and AR‐V7 using tumour tissues harvested from xenograft model mice (Figure 2F). IHC of tumour tissues from the negative control group showed that the expression of NSUN2 was correlated with the expression of AR (r2 = 0.4667, *p* = .0050) (Figure 2G) and AR‐V7 (r2 = 0.2800, *p* = .0425) (Figure 2H). The IHC scores of AR (Figure 2I) and AR‐V7 (Figure 2J) were markedly decreased in the NSUN2 shRNA group and increased in the NSUN2 OE group, indicating that NSUN2 significantly regulates the expression of AR and AR‐V7. Decreased tumour volume and weight were observed in the NSUN2 shRNA group, and increased tumour size was observed in the NSUN2 OE group compared with the control group. Tumour volume (Figure 2K) and weight (Figure 2L) were markedly decreased in the shRNA‐treated group and increased in the NSUN2 OE group compared with the negative control group. In conclusion, NSUN2 could influence the proliferation, invasion and migration of PCa cells both in vitro and in vivo. NSUN2 has a significant positive correlation with AR and AR‐V7 expression.

### 3.3 Silencing NSUN2 decreased m^5^C levels and downregulated AR expression and signalling activity

**FIGURE 3:**
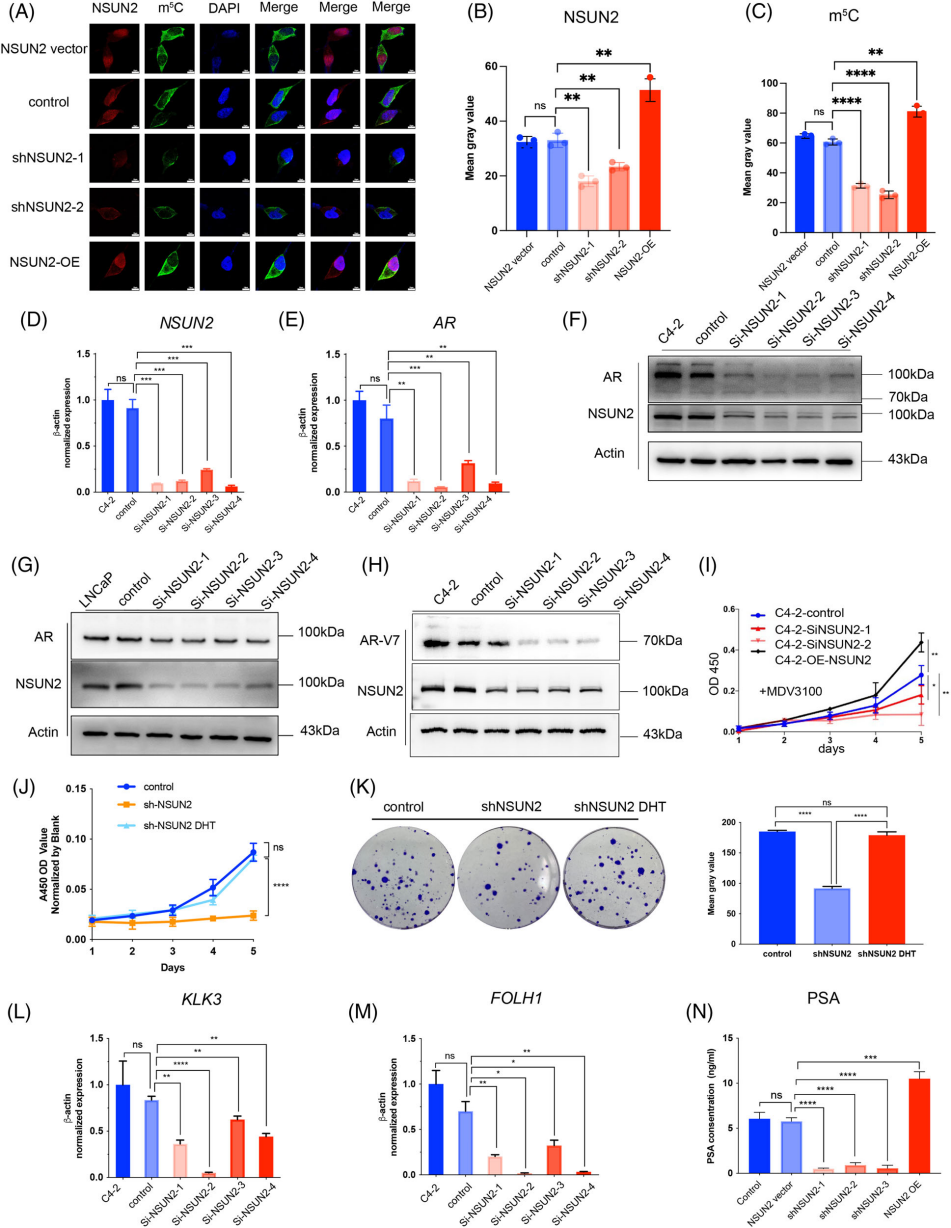
Knockdown of NSUN2 reduces the m^5^C level and decreases AR expression and activity. (A–C) Immunocytochemical staining and cell imaging were performed to detect the NSUN2 and m^5^C levels in C4‐2 cells with or without NSUN2 knockdown or OE. (*n* = 3 independent experiments). The *p* values were determined using a two‐sided unpaired student's *t*‐test. D‐E qPCR analysis of the mRNA levels of AR (D) and NSUN2 (E) in C4‐2 cells with or without NSUN2 knockdown. (F,G) Immunoblot analysis of AR, NSUN2 and β‐actin expression in C4‐2 (F) and LNCaP (G) cells with or without NSUN2 knockdown. (H) Immunoblot of AR‐V7 and NSUN2 in C4‐2 cells with or without NSUN2 knockdown. (I) CCK‐8 assay of MDV3100 in C4‐2 cells with or without NSUN2 knockdown or NSUN2 OE. (*n* = 3 independent experiments). The *p* values were determined using a two‐sided unpaired student's *t*‐test. (J) CCK‐8 assay of DHT in LNCaP cells with or without NSUN2 knockdown (shNSUN2‐2 was used). (*n* = 3 independent experiments). The *p* values were determined using a two‐sided unpaired student's *t*‐test. (K) Colony formation assay of LNCaP cells with or without NSUN2 knockdown (shNSUN2‐2 was used) treated with DHT (*n* = 3 independent experiments). The *p* values were determined using a two‐sided unpaired student's *t*‐test. L‐M qPCR analysis of the mRNA levels of KLK3 (L) and FOLH1 (M) in C4‐2 cells with or without NSUN2 knockdown or OE. (N) Detection of PSA levels in C4‐2 cells with or without NSUN2 knockdown or OE by immunofluorescence (*n* = 3 independent experiments). The *p* values were determined using a two‐sided unpaired student's *t*‐test

NSUN2 normally functions as a methyltransferase that catalyses the methylation of cytosine to 5‐methylcytosine at RNA. Subcutaneous xenografts IHC results showed that NSUN2 is co‐expressed with AR and AR‐V7. The immunofluorescence assay in NSUN2 knockdown or overexpression C4‐2 cells showed that the m^5^C fluorescence signal was significantly positively correlated with NSUN2 expression (all *p* < .05, Figure 3A‐C). These results indicated that NSUN2 was responsible for the m^5^C modification level in C4‐2 cells. Consistently the *AR* mRNA level was decreased when NSUN2 was downregulated by siRNAs (Figure 3D,E). Additionally, silencing NSUN2 led to a decrease in AR protein levels in both C4‐2 and LNCaP cells (Figure 3F,G). AR‐V7 is encoded by a transcript variant of *AR* with different splicing at the pre‐mRNA level and was reported to influence enzalutamide resistance in PCa.^12^ We found that the protein level of AR‐V7 was downregulated after knockdown of NSUN2, suggesting that NSUN2 may regulate *AR* at the pre‐mRNA level (Figure 3H). We designed primers at each intron of *AR* pre‐mRNA (Figure S3A) and found each intron of *AR* pre‐mRNA expression was decreased while NSUN2 was silenced (Figure S3B–H). Furthermore, we found that the expression level of NSUN2 could affect the tolerance of C4‐2 cells to enzalutamide (MDV3100) (Figure 3I). At the same time, the proliferation and migration ability of 22RV1 cells were also affected by the expression level of NSUN2 (Figure S3 I–K). Dihydrotestosterone (DHT) can promote the development of the prostate and accelerate the AR transfer into the nucleus, functioning as a transcription factor. Through CCK‐8 (Figure 3J) and clone formation assays (Figure 3K) with NSUN2 knockdown LNCaP cells treated with DHT, we found that DHT can rescue the NSUN2 expression (Figure S3L) and the cell proliferation ability of NSUN2 knockdown cells. Therefore, NSUN2‐mediated regulation of PCa progression may be dependent on AR signalling.

To detect whether the AR signalling activity was also influenced by NSUN2 expression, we detected *KLK3* (known as prostate‐specific antigen [PSA]) and *FOLH1* (known as prostate‐specific membrane antigen [PSMA]) at the transcriptional level by quantitative real‐time PCR (RT‐qPCR). The transcriptional levels of *KLK3* and *FOLH1* were decreased by siRNA‐mediated silencing of NSUN2 (Figure 3L,M). The PSA protein levels in the C4‐2 cell supernatant were changed along with NSUN2 expression, as indicated by ELISA (Figure 3N).

### 3.4 NSUN2‐mediated m^5^C modifications are clustered at the 5′ end of *AR* mRNA

**FIGURE 4:**
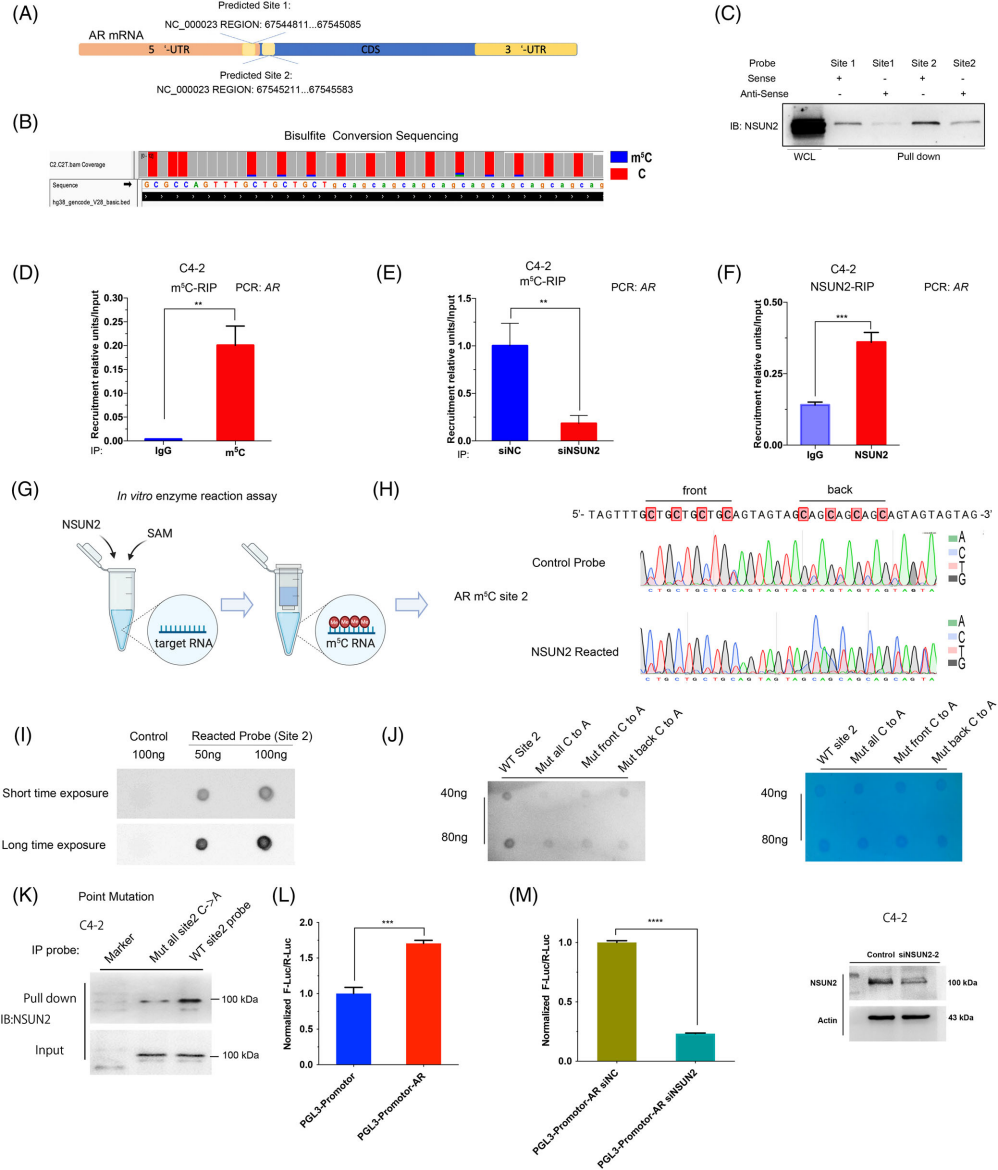
NSUN2 mediates the m^5^C modification of AR mRNA. (A) Diagram of the two predicted m^5^C sites in AR mRNA. (B) Modification site 2 identified by RNA‐BisSeq of mRNA from C4‐2 cells. (C) Synthetic RNA probes of AR m^5^C modification regions pulled down the NSUN2 protein. (D) Assessment of the m^5^C modification of AR mRNA by m^5^C‐RIP qPCR (*n* = 3 independent experiments). The *p* values were determined using a two‐sided unpaired student's *t*‐test. (E) Assessment of the m^5^C modification of AR mRNA by m^5^C‐RIP qPCR in C4‐2 cells with or without NSUN2 knockdown (siNSUN2‐2 was used) (*n* = 3 independent experiments). The *p* values were determined using a two‐sided unpaired student's *t*‐test. (F) Identification of binding sites between the NSUN2 protein and AR mRNA by NSUN2‐RIP qPCR (*n* = 3 independent experiments). The *p* values were determined using a two‐sided unpaired student's *t*‐test. (G,H) Identification of the specific m^5^C modification site through Sanger sequencing of bisulphite conversion probes modified by an in vitro RNA m^5^C transferase reaction. (I) Dot blot detection of AR m^5^C modification site 2 by in vitro RNA m^5^C transferase reaction. (J) Dot blot detection of the m^5^C modification level of the wild‐type or mutated AR probes modified by in vitro RNA m^5^C transferase reaction. Methylene blue was used as a loading control. (K) Pull‐down assay with wild‐type or mutated probes to confirm the NSUN2 binding site for AR mRNA. (L) Relative luciferase activity of the luciferase reporter gene, with or without the AR modification region in C4‐2 cells. Each well (∼10^6^ cells) was transfected with 2 µg luciferase reporter plasmid and 1 µg pRL‐TK plasmid (Renilla luciferase reporter). (M) Relative luciferase activity of the luciferase reporter gene containing the AR modification region in C4‐2 cells with or without NSUN2 knockdown (siNSUN2‐2 was used). Each well (∼10^6^ cells) was transfected with 2 µg luciferase reporter plasmid and 1 µg pRL‐TK plasmid (Renilla luciferase reporter) (*n* = 3 independent experiments). The *p* values were determined using a two‐sided unpaired student's *t*‐test. Immunoblot detection of NSUN2 and β‐actin expression in C4‐2 cells with or without NSUN2 knockdown (siNSUN2‐2 was used)

Given that NSUN2 silencing attenuated the expression of AR at both the transcriptional and protein levels, and AR signalling activity, we hypothesized that m^5^C modification may exist in *AR* mRNA. We performed RNA bisulphite sequencing (RNA‐BisSeq) in C4‐2 cells and observed multiple unconverted cytosines near the end of the 5′‐UTR and start codon of *AR* (Figure 4A,B). We called the m^5^C cluster near the end of the 5′‐UTR 'site 1' and the m^5^C cluster near the start codon 'site 2'. We next performed the pull‐down assays with synthesized probes of site 1 and site 2. The results showed that both site 1 and site 2 RNA could bind to the NSUN2 protein in C4‐2 cells. Since the site 2 m^5^C signals in the BisSeq and pulldown assays were stronger than those for site 1, the following experiments were conducted using site 2 (Figure 4C). RNA immunoprecipitation (RIP) assays showed that the m^5^C antibody could enrich *AR* mRNA in vitro (Figure 4D), whereas after silencing NSUN2, less *AR* mRNA was enriched in the m^5^C‐RIP assay (Figure 4E). The NSUN2‐RIP assay showed that the NSUN2 antibody group could markedly enrich *AR* mRNA in C4‐2 cells compared with the IgG group (Figure 4F). To test the reliability of the above experiment, we performed the same test with a known *HDGF* m^5^C modification site^17^ (Figure S4A,B). Previous reports discussed only the NSUN2 mononucleotide activity as an mRNA methyltransferase.^30^ To test whether NSUN2 is able to modify m^5^C of *AR* mRNA in clusters, we carried out the in vitro enzyme reaction assays using in vitro‐transcribed fragments of *AR* mRNA, recombinant NSUN2 protein, and S adenosyl‐L‐methionine (SAM) (Figure 4G). After the enzyme reaction, the probes of site 2 were examined by Sanger sequencing upon bisulphite conversion, and we found multiple C signals for NSUN2‐reacted probes that were stronger than those for the control probes (Figure 4H). The dot blot assay was conducted to confirm the reaction assay. The reacted probes could enrich the m^5^C antibodies, but the untreated control probes could not (Figure 4I). In order to assess that the clustered C sites on AR mRNA are critical for the enzymatic activity of NSUN2, we mutated the first four Cs and the last four Cs individually or all. An in vitro enzyme assay demonstrated that mutation of multiple C sites decreased the m^5^C level of *AR* site 2 RNA probes (Figure 4J). Mutated and wild‐type site 2 probes were used in the pulldown assay and found that site 2 cytosines were found to be crucial for NSUN2 binding (Figure 4K). Finally, the *AR* site 2 sequence was inserted into the PGL3‐promoter plasmid. The group with the *AR* site 2 sequence showed stronger luciferase activity than the control group (Figure 4L) and was dependent on the presence of NSUN2 (Figure 4M).

### 3.5 YBX1 is the reader of *AR* mRNA m^5^C modification

**FIGURE 5:**
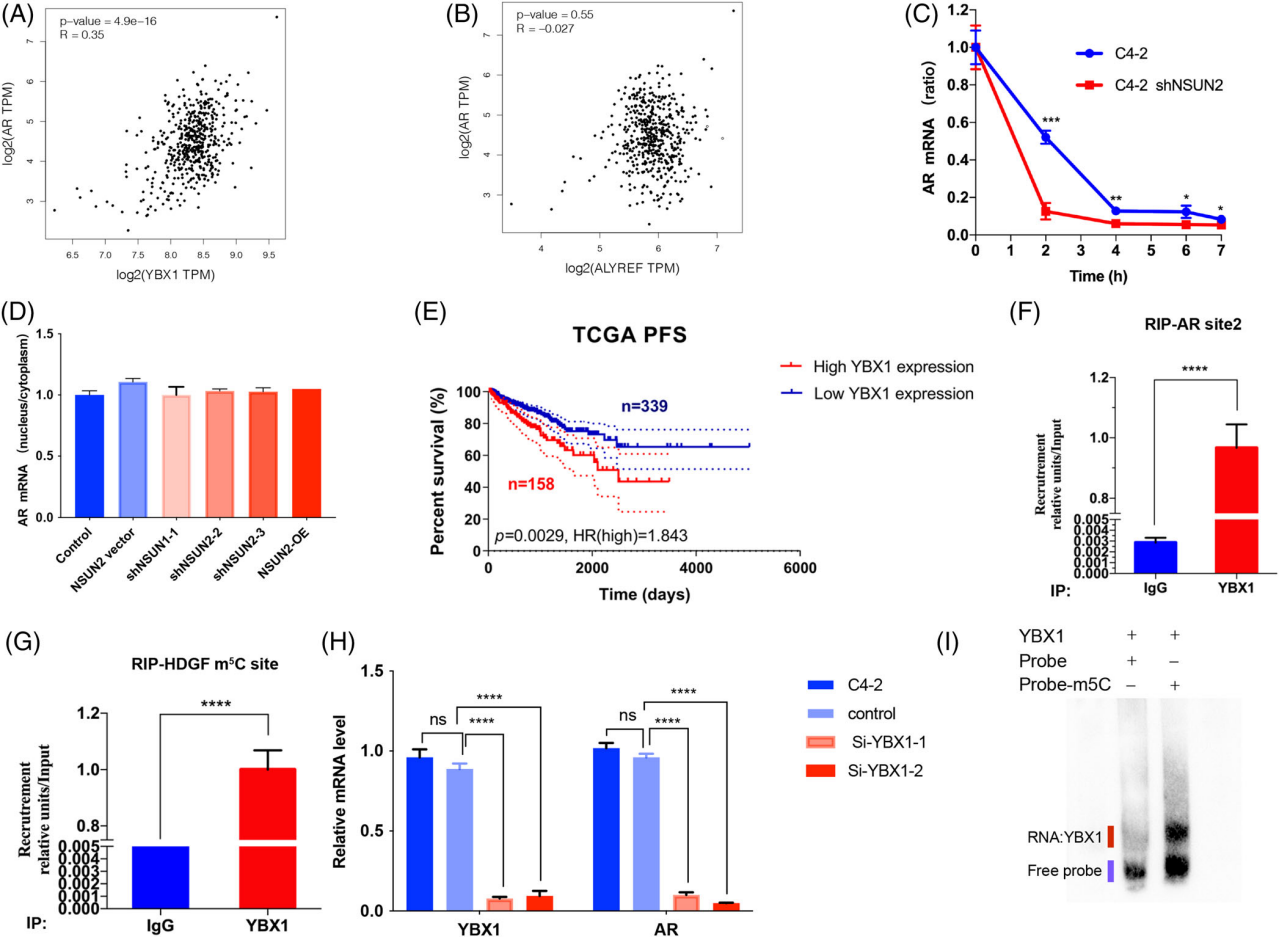
YBX1 is the reader of AR mRNA m^5^C sites. (A) Scatterplot of YBX1 expression and AR expression in the TCGA cohort. (B) Scatterplot of ALYREF expression and AR expression in the TCGA cohort. (C) qPCR analysis of the mRNA stability of AR, shNSUN2‐2 was used in the shNSUN2 group. (D) Nuclear and cytoplasmic mRNA were extracted, and AR mRNA was detected by qRT‐PCR in C4‐2 cells with or without NSUN2 knockdown or OE. (E) K‐M plot of the disease‐free survival of PCa patients with high or low YBX1 expression in the TCGA cohort. (F) Assessment of the binding abilities of YBX1 with AR mRNA in C4‐2 cells by YBX1‐RIP qPCR (*n* = 3 independent experiments). The *p* values were determined using a two‐sided unpaired student's *t*‐test. (G) Assessment of the HDGF m^5^C modification sites in C4‐2 cells by YBX1‐RIP qPCR (*n* = 3 independent experiments). The *p* values were determined using a two‐sided unpaired student's *t*‐test. (H) Silencing YBX1 in C4‐2 cells decreased AR mRNA expression (*n* = 3 independent experiments). The *p* values were determined using a two‐sided unpaired student's *t*‐test. (I) Assessment of the ability of YBX1 protein (200 ng) to bind with modified (the right line) or unmodified (the left line) AR site 2 probes (20 ng) by EMSA (*n* = 3 independent experiments)

To investigate the recognition mechanism of *AR* mRNA m^5^C modification, we first analysed the already known m^5^C 'readers,' YBX1 and ALYREF. As reported, ALYREF participates in mRNA export and YBX1 plays a role in mRNA stability.^17^, ^18^ A scatter plot of *YBX1* expression and *AR* expression in the TCGA PRAD cohort (*N* = 497) indicated that *YBX1* expression was positively correlated with *AR* expression (*p* = 4.9e‐16, Figure 5A). However, *ALYREF* expression was not correlated with *AR* expression (*p* = .55, Figure 5B). Silencing NSUN2 influenced *AR* mRNA stability (Figure 5C) but did not influence RNA export from the nucleus to the cytoplasm (Figure 5D). Clinically, elevated *YBX1* expression correlated with shortened PFS in 497 patients from the TCGA cohort (*p* = .0029, HR = 1.843, Figure 5E). RIP assays showed that YBX1 could bind *AR* mRNA (Figure 5F) and *HDGF* was used as a positive control (Figure 5G). Additionally, silencing YBX1 in C4‐2 cells led to decreased *AR* mRNA (Figure 5H). The luciferase activity was decreased when knockdown YBX1 (Figure S4C). This may not be related to the transcriptional activity of YBX1, because YBX1 could not be a transcription factor of *AR* (Figure S4D). Furthermore, we used an electrophoretic mobility shift assay (EMSA) to assess the binding abilities of the YBX1 protein with m^5^C modified or unmodified *AR* site 2 probes (20 ng). YBX1 could only bind m^5^C‐modified probes (Figure 5I). The pulldown assay came to the same conclusion (Figure S4E).

### 3.6 AR regulates m^5^C and NSUN2 in PCa

**FIGURE 6:**
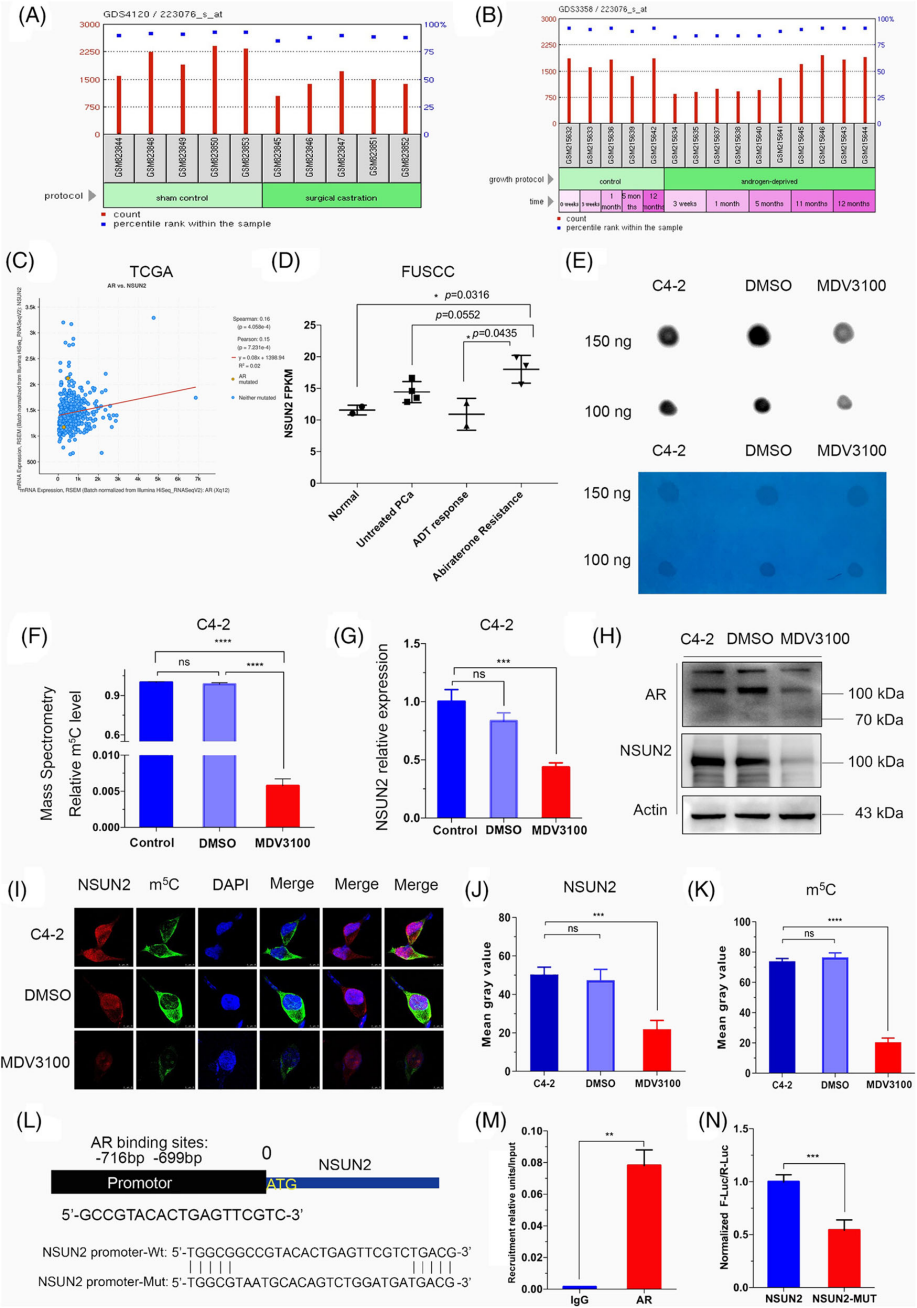
AR inhibition decreases the mRNA m^5^C level and downregulates NSUN2 expression. (A) GEO database (GDS4120) analyses the NSUN2 expression after surgical castration. (B) GEO database (GDS3358) analyses NSUN2 expression after androgen deprivation. (C) Scatterplot of NSUN2 expression and AR expression in the TCGA cohort. (D) NSUN2 expression was detected by RNA‐Seq in normal prostate (radical cystectomy sample), untreated PCa, ADT‐responsive (3‐month ADT‐treated), and abiraterone‐resistant PCa (PSA progression after abiraterone) samples from the FUSCC cohort. (E) m^5^C dot blot assay using poly(A)+ mRNA of C4‐2 cells treated with or without enzalutamide. The experimental group was treated with 50 nM enzalutamide for 72 h. Methylene blue was used as a loading control (*n* = 3 independent experiments). (F) RNA m^5^C quantification by LC/MS/MS using poly(A)+ mRNA of C4‐2 cells treated with or without enzalutamide; the experimental group was treated with 50 nM enzalutamide for 72 h (*n* = 3 independent experiments). The *p* values were determined using a two‐sided unpaired student's *t*‐test. (G) qPCR analysis of the mRNA levels of NSUN2 in C4‐2 cells; the experimental group was treated with 50 nM enzalutamide for 72 h. (H) Immunoblot analysis of AR, NSUN2 and β‐actin expression in C4‐2 cells; the experimental group was treated with 50 nM enzalutamide for 72 h. (I–K) Immunocytochemical staining and cell imaging were performed to detect the NSUN2 and m^5^C levels in C4‐2 cells; the experimental group was treated with 50 nM enzalutamide for 72 h (*n* = 3 independent experiments). The *p* values were determined using a two‐sided unpaired student's *t*‐test. (L) Diagram of the NSUN2 promoter and the AR binding region. (M) ChIP assay confirmed the 19‐bp AR binding site in the NSUN2 promoter (*n* = 3 independent experiments). The enrichment abundance of each group was normalized by the input value. The *p* values were determined using a two‐sided unpaired student's *t*‐test. (N) Mutation of the AR binding site decreased the promoter activity in the luciferase reporter assay (*n* = 3 independent experiments). Each well (∼10^6^ cells) was transfected with 2 µg luciferase reporter plasmid and 1 µg pRL‐TK plasmid (Renilla luciferase reporter). The *p* values were determined using a two‐sided unpaired student's *t*‐test. ADT, androgen deprivation therapy; PCa, prostate cancer

In Gene Expression Omnibus (GEO) dataset (GDS4120), we found that *NSUN2* expression levels in mice LuCaP35 xenografts decreased after 4 weeks of surgical castration^31^ (Figure 6A). In the GDS3358 dataset, *NSUN2* expression levels in androgen‐deprived LNCaP decreased from 3 weeks to 5 months and rebounded in 11 months^32^ (Figure 6B). Notably, *AR* expression was positively correlated with the *NSUN2* level in the TCGA PRAD dataset (Figure 6C). In FUSCC PCa samples, *NSUN2* expression was greatly altered during the period of ADT. *NSUN2* expression decreased in an initial ADT for 3 months, whereas abiraterone‐resistant PCa samples showed markedly higher expression of *NSUN2* (Figure 6D). These analyses together suggest that AR may play an important role in regulating NSUN2, thereby the formation of m^5^C RNA. MDV3100 (enzalutamide) is a newly developed AR antagonist.^10^ We used enzalutamide to block AR activity in C4‐2 cells for 72 h and found that the m^5^C levels were dramatically decreased in a dot blot assay (Figure 6E). Consistently, RNA m^5^C quantification by LC/MS/MS showed similar results in C4‐2 cells (Figure 6F, Figure S4F,G). Interestingly, the expression of NSUN2 and AR decreased in C4‐2 cells upon enzalutamide treatment (Figure 6G,H) and apalutamide treatment (Figure S5A,B). We next conducted a fluorescence immunocytochemical staining assay and performed a live cell imaging confocal microscopy analysis. The results showed that the NSUN2 signal was mainly distributed in the nucleus, while the m^5^C signal was mainly distributed in the cytoplasm of C4‐2 cells (Figure 6I). Enzalutamide treatment significantly decreased NSUN2 expression and m^5^C levels (Figure 6J,K). In xenograft mouse model, shNSUN2 or treatment with apalutamide can inhibit tumour proliferation, and the combination of shNSUN2 and apalutamide is more effective (Figure S5C–E). These results support that AR inhibition downregulates NSUN2 expression and decreases the mRNA m^5^C level. The combination of shNSUN2 and AR inhibitors may become an effective clinical treatment.

We further investigated whether AR directly regulates *NSUN2* transcription. So, we predicted the AR DNA‐binding motifs in the *NSUN2* promoter sequences using the JASPAR database (http://jaspar.genereg.net).^33^ The AR binding site was revealed to be a 19‐bp sequence, approximately 700 bp upstream of the transcriptional start site (TSS) of *NSUN2* (Figure 6L). ChIP‐qPCR assays also showed significant enrichment of AR in the *NSUN2* promoter region (*p* < .05, Figure 6M). To further validate the binding site, we constructed a luciferase plasmid with wild‐type and mutated AR binding sites in the *NSUN2* promoter. AR binding site was verified by dual‐luciferase analysis (*p* < .01, Figure 6N). Apalutamide can decrease luciferase activity (Figure S5F). ChIP‐seq for H3K27ac profiling of PCa samples showed that the promoter region of *NSUN2* was active in PCa samples ^34^, ^35^(Figure S6A). Chromatin immunoprecipitation sequencing (ChIP‐seq) profiling of AR genome‐wide binding sites demonstrated that the *NSUN2* promoter region has significant AR binding signals in both PCa cell lines (Figure S6B) and tumour samples (Figure 6C).

### 3.7 NSUN2 is positively correlated with AR signalling genes and predicts poor outcomes in PCa

**FIGURE 7:**
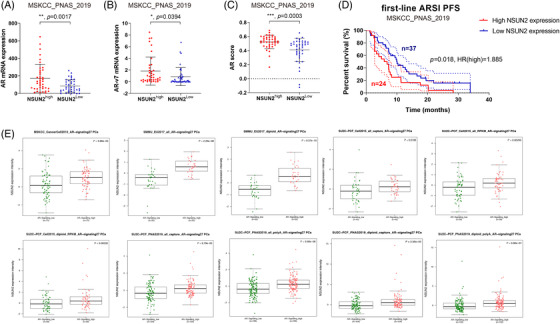
NSUN2 expression is positively correlated with the expression of AR signalling‐related genes and predicts PFS in multiple cohorts. (A–C) AR (A) and AR‐V7 (B) mRNA expression and AR score (C) were plotted for 91 PCa patients from the MSKCC cohort stratified according to NSUN2 expression. (D) The Kaplan–Meier (K–M) curve of first‐line ARSI PFS was plotted for the MSKCC cohort. (E) Scatter diagrams of NSUN2 mRNA expression in high or low AR signaling from MSKCC, SMMU, SU2C‐PCF cohorts. PCa, prostate cancer

**FIGURE 8:**
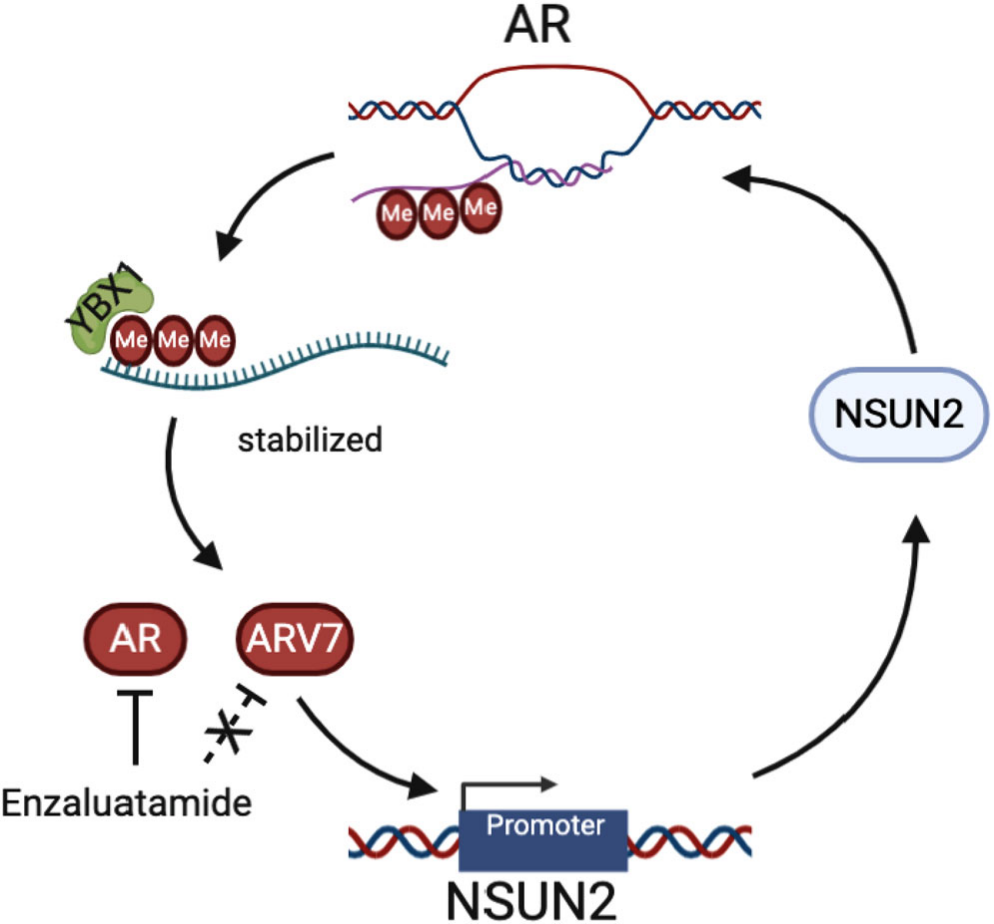
Model of the AR and NSUN2 positive feedback loop. AR pre‐mRNA is modified by NSUN2 and recognized by YBX1 and maintains its stability. At the same time, AR can act as a transcription factor to regulate the expression of NSUN2. In this way, a positive feedback loop is formed between NSUN2 and AR to promote the progression of prostate cancer. AR inhibitors can inhibit the transcriptional capacity of AR, but not AR alternative spliceosomes, such as AR‐V7. NSUN2 can regulate the stability of AR, and inhibition of NSUN2 in combination with AR inhibitors may better inhibit the progression of prostate cancer

To evaluate the expression patterns and prognostic values of NSUN2 in PCa, external validation cohorts were analysed. Significantly elevated *AR* (Figure 7A) and *AR‐V7* (Figure 7B) mRNA expression and AR scores (Figure 7C) were found in the high *NSUN2* expression group compared with the low *NSUN2* expression group among 91 PCa patients from the Memorial Sloan Kettering Cancer Center (MSKCC) cohort^8^ (*p* < .05). Most of these patients received first‐line second‐generation androgen receptor signalling inhibitor (ARSI) treatment. The progression‐free survival (PFS) after ARSI was plotted according to the level of *NSUN2* expression. High *NSUN2* expression significantly predicted poor PFS for 61 PCa patients from the MSKCC cohort (*p* = .018, HR = 1.885, Figure 7D). We analysed data for 598 PCa patients from multiple cohorts and found that increased *NSUN2* mRNA expression was significantly correlated with elevated AR signalling scores ^8^, ^15^, ^36^, ^37^ (Figure 7E). In conclusion, NSUN2 can influence the expression of AR and affect the effect of ARSI treatment. On the other hand, AR also had some impact on NSUN2 expression (Figure 8).

## 4 DISCUSSION

The role of mRNA m^5^C modification in cancers is largely unknown.^17^ Previous studies indicated that dynamic m^5^C modifications are associated with testis development in mice.^18^, ^21^ Testis development is dependent on AR expression and the AR signalling pathway. Also, AR is an important therapeutic target in PCa management. In addition, investigators have found that the signal of m^5^C modifications decreased in enzalutamide‐treated C4‐2 cells, so we tried to uncover the functions of m^5^C modifications in PCa. Our data provide evidence that NSUN2 is highly expressed in PCa and is associated with poor prognosis. Mechanistically, NSUN2 post‐transcriptionally stabilized *AR* by cluster m^5^C modification in an m^5^C‐YBX1‐dependent manner. At the same time, AR acts as a transcription factor to regulate the transcription of *NSUN2*. The positive feedback between NSUN2 and AR provides a possible model to explain PCa progression and the occurrence of CRPC.

RNA modifications like m^6^A have been reported to regulate diverse cellular functions in many tumours.^38^ Recently, the mRNA m^5^C modification has been identified to regulate mRNA metabolism and translocation.^17^, ^18^ NSUN2 and NSUN6 are the two known m^5^C methyltransferases (‘writers’) of mRNA to date.^18^, ^20^, ^21^ However, we found only NSUN2 can be the poor outcome predictor of PCa. NSUN2‐related m^5^C modification sites on mRNA are generally located in the translational start sites, 3′ untranslated regions (UTRs).^39^, ^40^ We found the m^5^C sites were clustered in the 5′ end of *AR* mRNA and maintains the stability of *AR* mRNA. Traditional analysis of m^5^C modification sites used to ignore the cluster C signal for two reasons. First, false positives may occur due to incomplete bisulphite conversion in the GC‐rich sequence, which more easily forms secondary structures.^41^, ^42^ Second, the validation of the mono m^5^C site by point mutation is much easier than that of cluster m^5^C sites. In this study, we applied multiple experiments to illustrate that *AR* mRNA has a cluster of m^5^C modifications in the 5′‐end regions. We used Sanger sequencing and dot blot methods to illustrate this phenomenon. This study provides novel evidence that m^5^C modification clusters exist and have functions. Previously, a study reported that NSUN2 stabilizes *CDKN2B/p16* mRNA.^43^ However, p16 is an inhibitor of CDK. It seems that NSUN2 would rather lead to reduce cell proliferation through p16. This contradicts the conclusion in this study that NSUN2 promotes PCa cell proliferation. Then, we found that the expression of NSUN2 was more strongly associated with AR than with p16 in PCa (Figure S7A). When NSUN2 was knocked down in C4‐2 cells, the expression level of AR was significantly reduced, while the change of p16 was not so obvious (Figure S7B). Therefore, we hypothesized that the effect of NSUN2 on p16 in PCa is not sufficient to influence NSUN2 to promote PCa cell proliferation through AR. At the same time, we found that high expression of *p16* in PCa is associated with poor DFS (Figure S7C,D). So, we predict that NSUN2 could promote PCa cell proliferation, mostly. Also, NSUN2 could methylate many other RNAs like from our data, such as FOXA1 and SMAD3 (Figure S7E–G). These genes could also contribute to the poor prognosis of PCa. Particularly, m^5^C added by NSUN2 has been detected not only in mRNA but also in rRNA and tRNA.^18^, ^39^ Recently, researchers have reported that NSUN3 is participating in the mitochondrial tRNA modifications and shaped metabolic plasticity in metastasis of oral cancer.^44^ It deserves further researches whether NSUN2 could influence the metabolism and oxidative stress through mitochondrial tRNAs.

Historically, ADT with or without first‐generation AR inhibitors such as flutamide and bicalutamide has been the standard of care for metastatic castration‐sensitive PCa.^45^ In this stage, the majority of patients have an initial response to ADT. However, most men with metastases develop CRPC in a median time frame of approximately one year.^4^, ^46^ We found NSUN2 expression levels followed the same trends in the previous studies^31^, ^32^ and our cohort. The success of the LATITUDE and ARCHES studies supports the hypothesis that more effective inhibition of AR signalling as the initial systemic therapy in patients with castration‐sensitive PCa leads to improved outcomes.^5^, ^9^, ^47^ However, metastatic PCa is still considered a lethal disease, and aberrations of the AR, steroidogenic parallel pathways, and neuroendocrine differentiation are the main reasons for resistance to next‐generation ADT.^11^, ^12^

AR‐V7 leads to ligand‐independent constitutive activation that is not inhibited by antiandrogen therapies. Among men with metastatic CRPC, the circulating tumour cell (CTC) AR‐V7+ rate was 8%.^48^ Previous reports suggested that CTC AR‐V7 detection is a poor prognostic indicator for the clinical efficacy of secondary hormone therapies, including abiraterone, enzalutamide or galeterone.^48^, ^49^ Outcome‐associated AR perturbations include AR variants and AR gene alterations.^3^ These aberrant AR products escape the regulation of any clinically available ARSIs. Our results suggest that *AR* pre‐mRNA can be modified by NSUN2 and maintained stable with the recognition and help of YBX1. Our study indicates NSUN2‐ m^5^C‐YBX1 axis could regulate ARV7 expression levels as well. Disruption of the NSUN2‐m^5^C‐YBX1 axis may interfere with the progression of CRPC due to AR variants and AR genomic alterations. This showed a novel mechanism that regulates all AR perturbations and may be therapeutic in AR‐related CRPC. However, there are currently no known specific inhibitors or antibodies for NSUN2 that can be used for clinical research. At present, there is a technology called PROTAC that uses the cell's own ubiquitination degradation mechanism to target and degrade specific proteins, which may be used for the inhibition of NSUN2, but further research is needed. As an important part of this regulatory axis, YBX1 may be an important target for PCa therapy. YBX1 has been reported to promote the progression of many kinds of cancers. Endogenous tRNA‐derived fragment displacement by YBX1 can suppress breast cancer progression.^50^ YBX1 acts as a reader to promote oestrogen resistance in progressive breast cancer cells, and can be inhibited by YBX1 phosphorylation inhibitor TAS0612 (a multi‐kinase inhibitor) and everolimus (a rapamycin complex 1 inhibitor).^51^ LncRNA can form a positive feedback loop with YBX1 to activate the FOXA1 transcription network in cancer.^25^ More importantly, FOXA1 is also an important transcription factor in PCa and is closely involved in the regulatory network of AR. Our data also shows that YBX1 can bind and stable the AR pre‐mRNAs which have been modified by m^5^C. Maybe, YBX1 is a good therapeutic target for PCa and even mCRPC.

In conclusion, we found that m^5^C modifications exist on *AR* mRNA. NSUN2 can methylate *AR* pre‐mRNA and influence *AR‐V7* expression, and YBX1 can bind and stable the m^5^C modified *AR* mRNA. AR can promote *NSUN2* transcription. The NSUN2 and AR feedback loop leads to a poor outcome in PCa due to ARSI resistance. These findings suggest NSUN2 and YBX1 as new targets for the treatment of PCa.

## CONFLICT OF INTEREST

The authors declare no conflict of interest.

## Supporting information

Supplementary Table 1: Clinicopathological parameters for 497 prostate cancer patients obtained from the Cancer Genome Atlas (TCGA).

Supplementary Table 2: Clinicopathological parameters for 88 high risk prostate cancer patients obtained from the Fudan University Shanghai Cancer Center (FUSCC).

Supporting Information

Supplementary Figure 1 Analysis of NSUN2 and NSUN6 in the TCGA PRAD cohort.

Supplementary Figure 2 Wound healing assay of C4‐2 and C4‐2R and LNCaP cells with NSUN2 knockdown or OE. Representative images at 0 and 36 h are presented.

Supplementary Figure 3 NSUN2 influenced AR expression and acted as an oncogene.

Supplementary Figure 4 Positive control of the m5C‐RIP assay and the LC/MS/MS original data.

Supplementary Figure 5 Apalutamide could decrease NSUN2 expression.

Supplementary Figure 6 ChIP‐seq data for AR and H3K27ac profiling.

Supplementary Figure 7 The action of NSUN2 on p16 does not affect its promotion of prostate cancer.

Supporting Information
