# Supplementary material for: Positive epigenetic regulation loop between AR and NSUN2 promotes prostate cancer progression
Source: Clin Transl Med. 2022 Sep 28;12(9):e1028. doi: 10.1002/ctm2.1028 (PMC9516604; doi:10.1002/ctm2.1028)
Supplement: Supplementary file 3 — Supporting Information [file CTM2-12-e1028-s011.docx]

Caption for supplementary table 1: Clinicopathological parameters for 497 prostate cancer patients obtained from the Cancer Genome Atlas (TCGA).

Caption for supplementary table 2: Clinicopathological parameters for 88 high risk prostate cancer patients obtained from the Fudan University Shanghai Cancer Center (FUSCC).
