# Supplementary figures and images for: Positive epigenetic regulation loop between AR and NSUN2 promotes prostate cancer progression
Source: Clin Transl Med. 2022 Sep 28;12(9):e1028. doi: 10.1002/ctm2.1028 (PMC9516604; doi:10.1002/ctm2.1028)

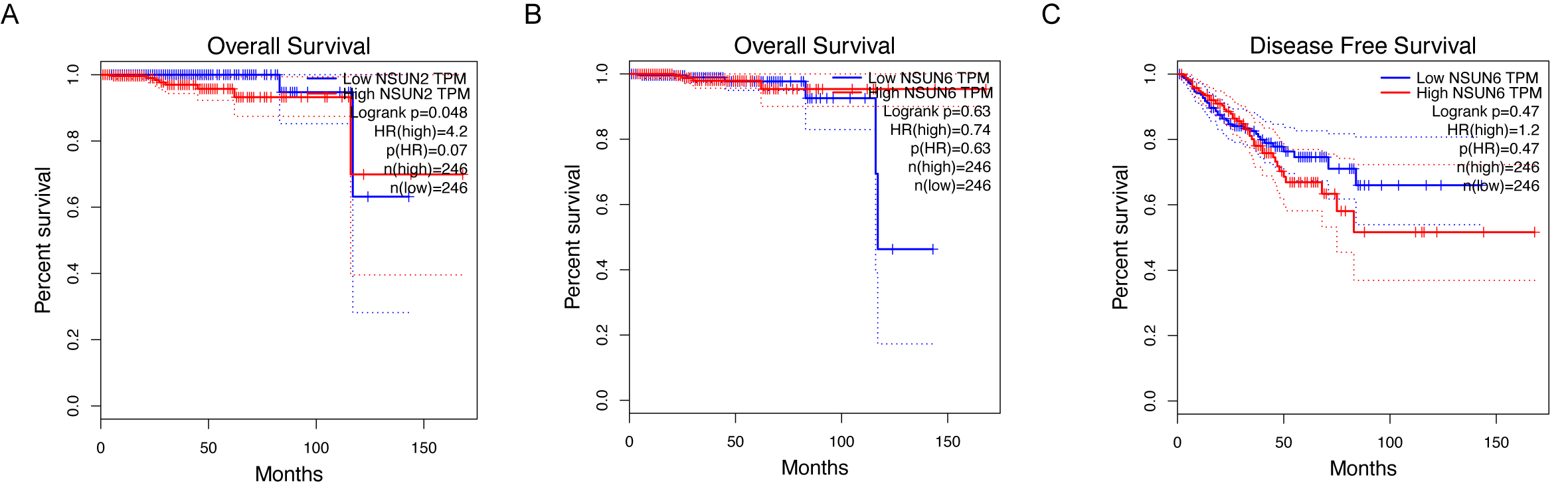

Supplement: Supplementary file 4 — Supplementary Figure 1 Analysis of NSUN2 and NSUN6 in the TCGA PRAD cohort. [file CTM2-12-e1028-s010.tif]

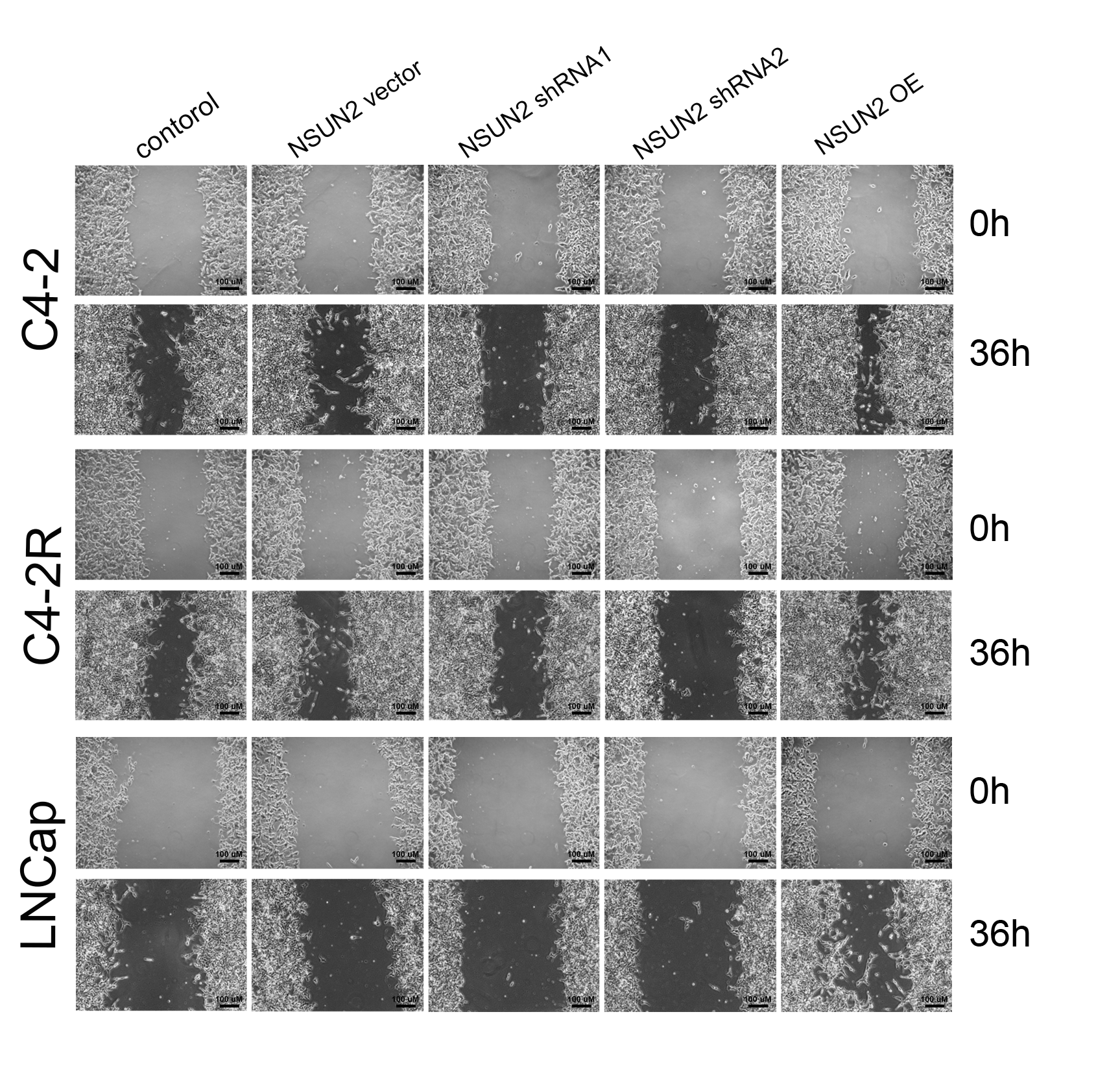

Supplement: Supplementary file 5 — Supplementary Figure 2 Wound healing assay of C4‐2 and C4‐2R and LNCaP cells with NSUN2 knockdown or OE. Representative images at 0 and 36 h are presented. [file CTM2-12-e1028-s003.tif]

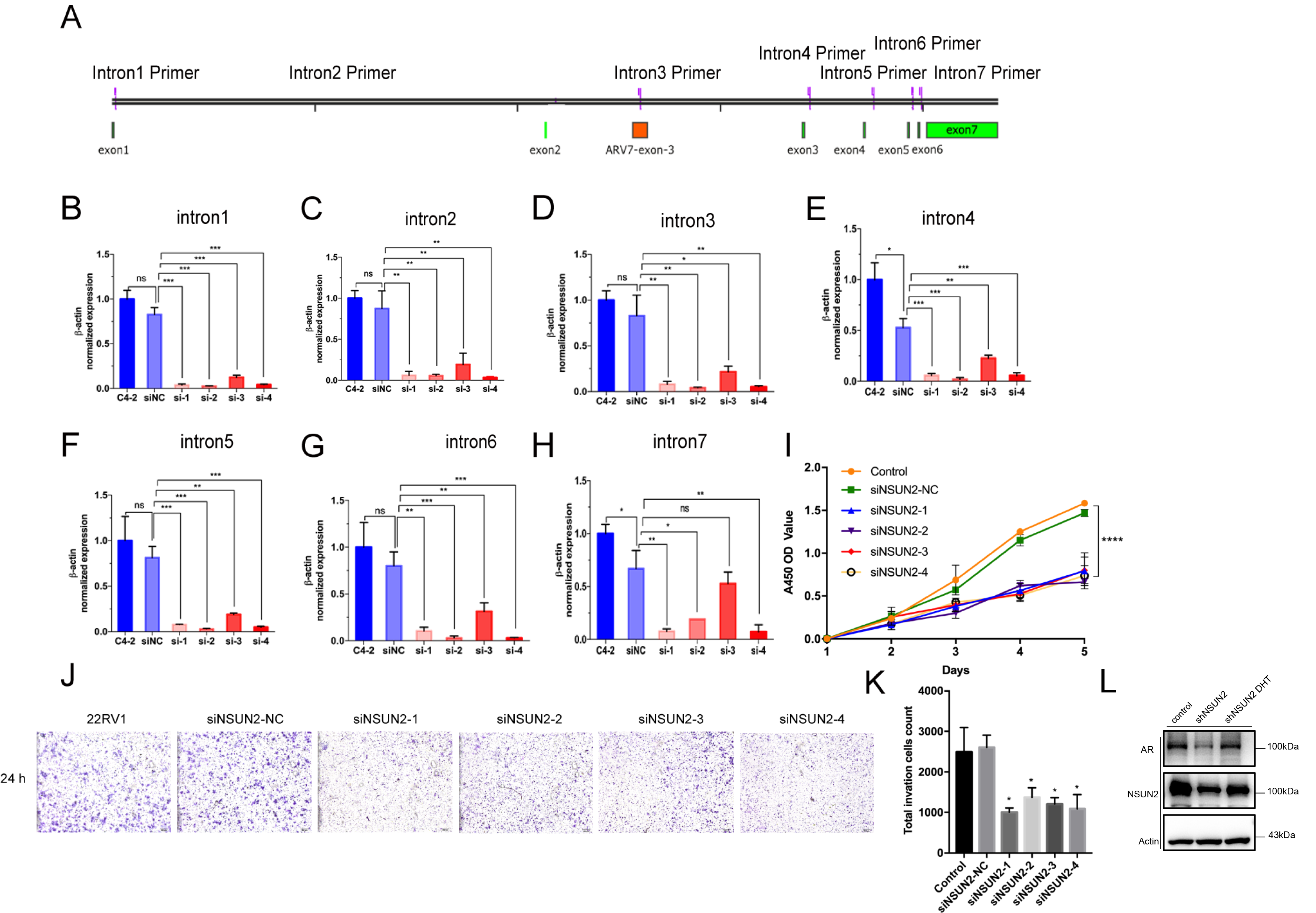

Supplement: Supplementary file 6 — Supplementary Figure 3 NSUN2 influenced AR expression and acted as an oncogene. [file CTM2-12-e1028-s004.tif]

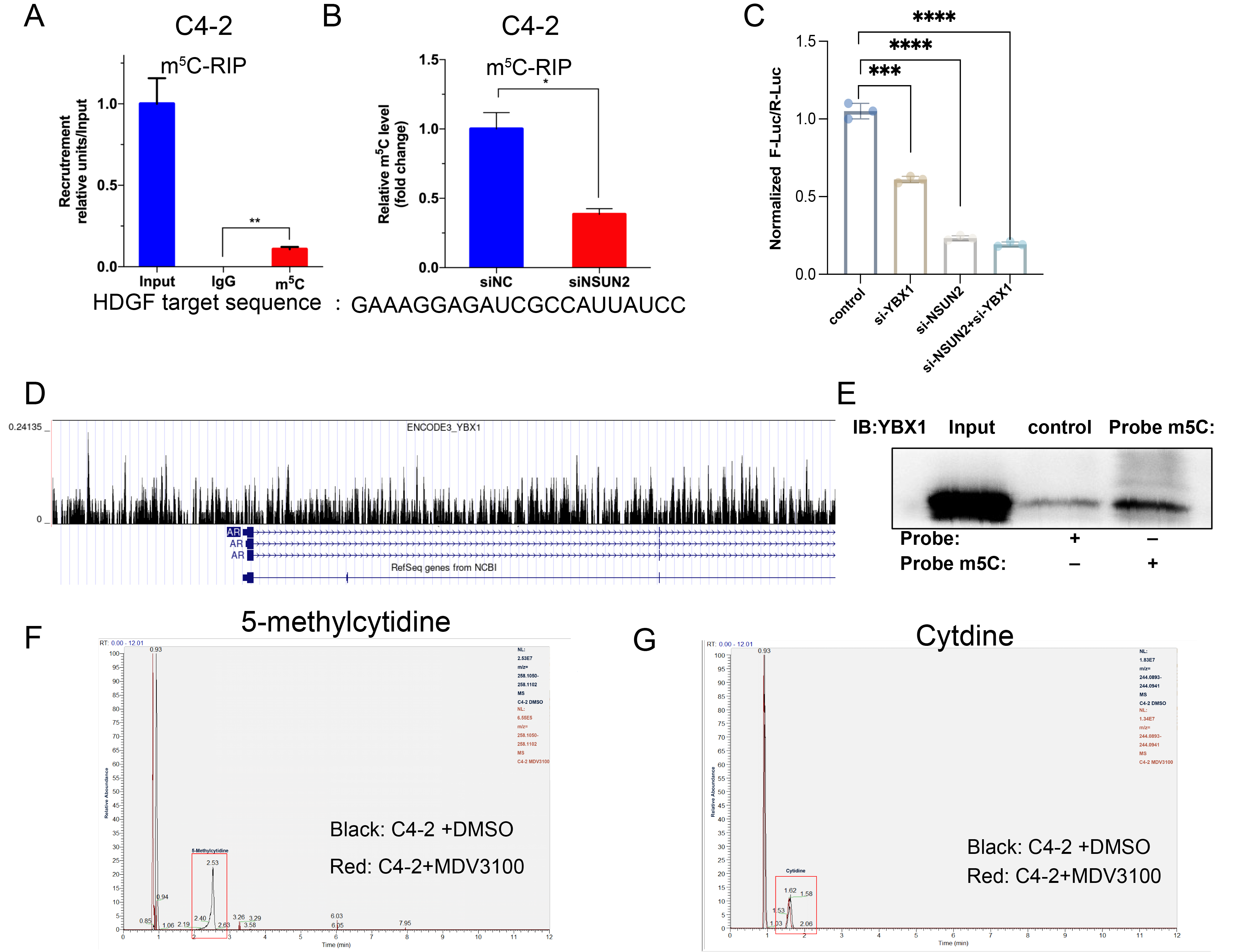

Supplement: Supplementary file 7 — Supplementary Figure 4 Positive control of the m5C‐RIP assay and the LC/MS/MS original data. [file CTM2-12-e1028-s006.tif]

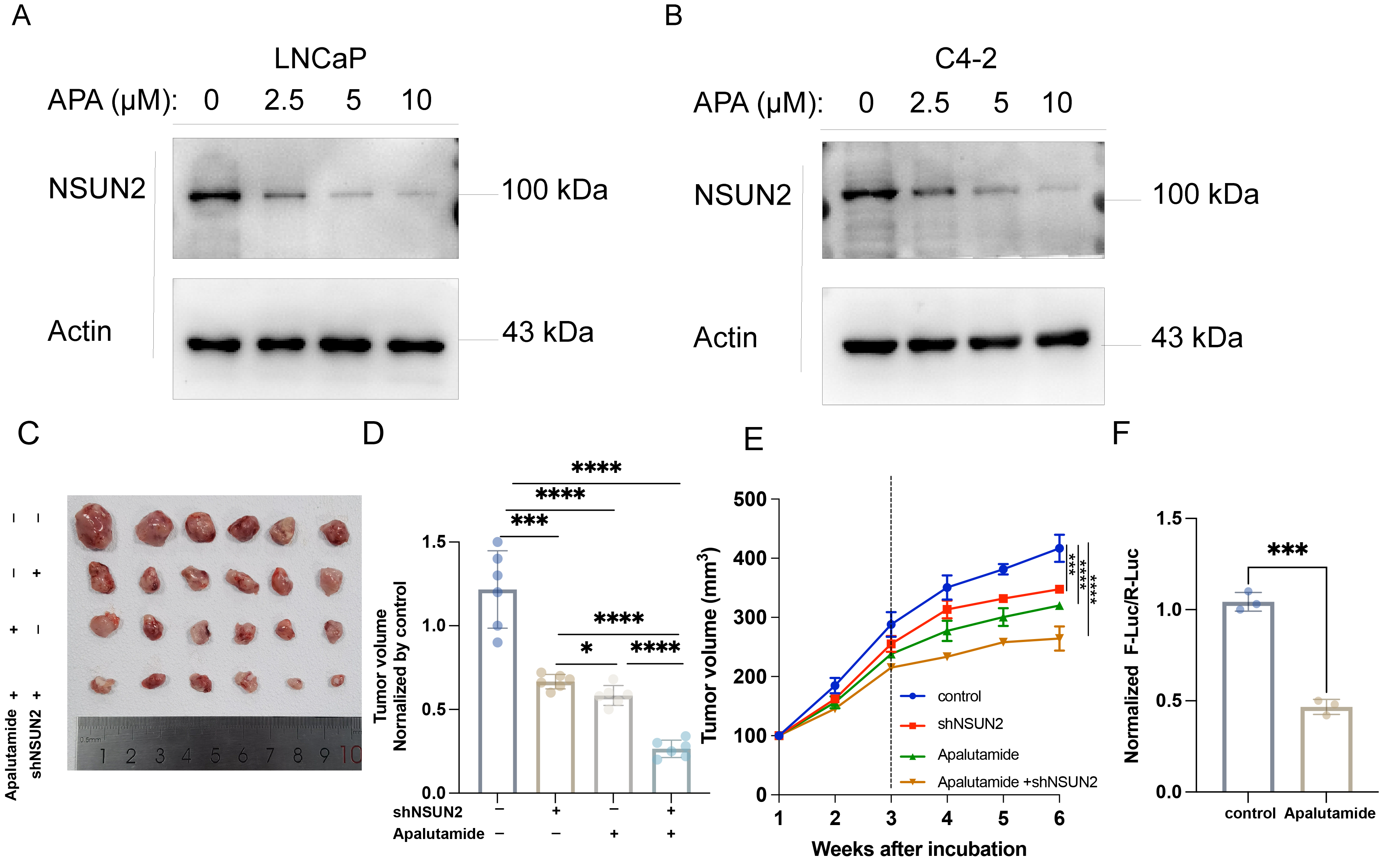

Supplement: Supplementary file 8 — Supplementary Figure 5 Apalutamide could decrease NSUN2 expression. [file CTM2-12-e1028-s002.tif]

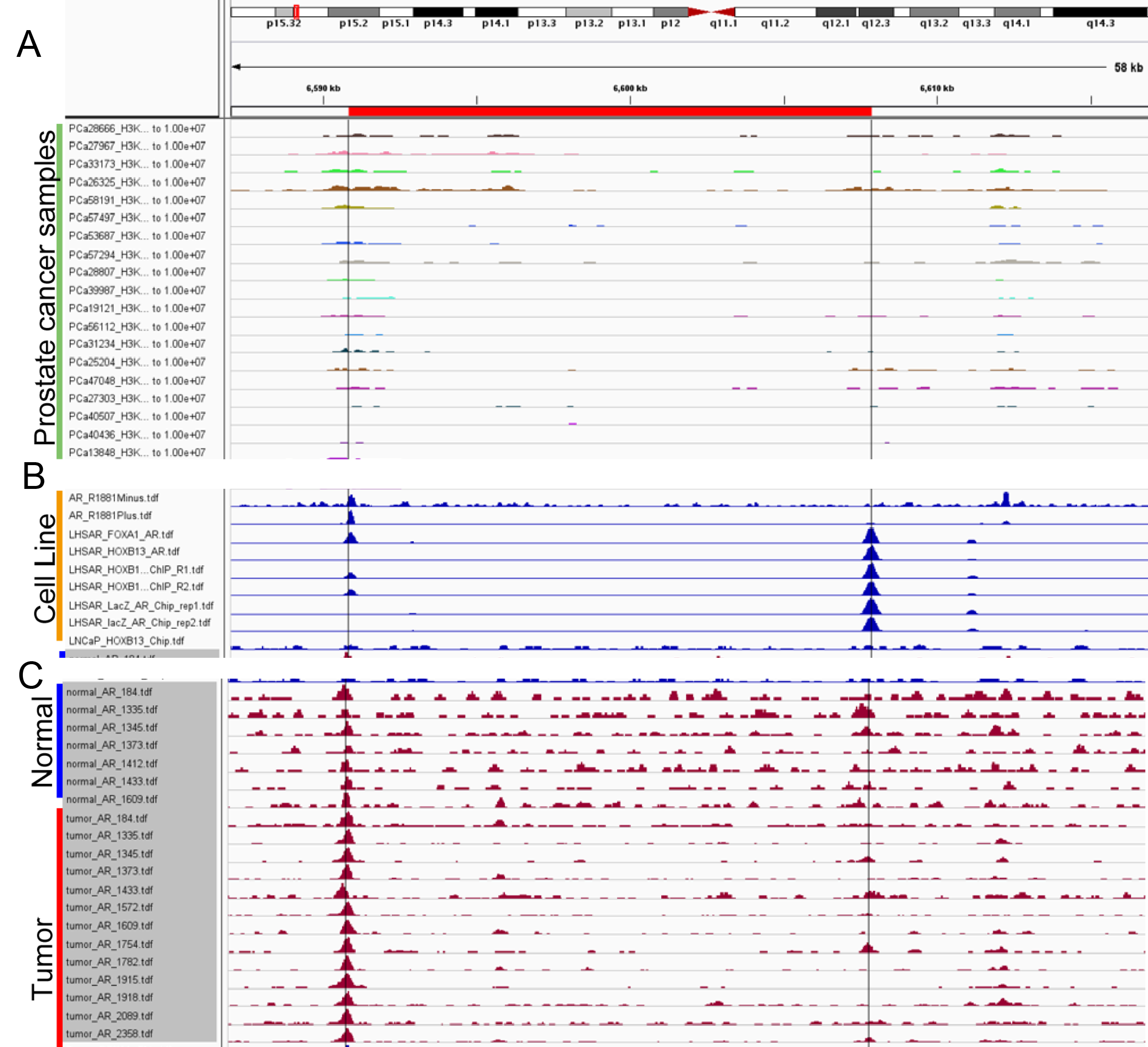

Supplement: Supplementary file 9 — Supplementary Figure 6 ChIP‐seq data for AR and H3K27ac profiling. [file CTM2-12-e1028-s009.tif]

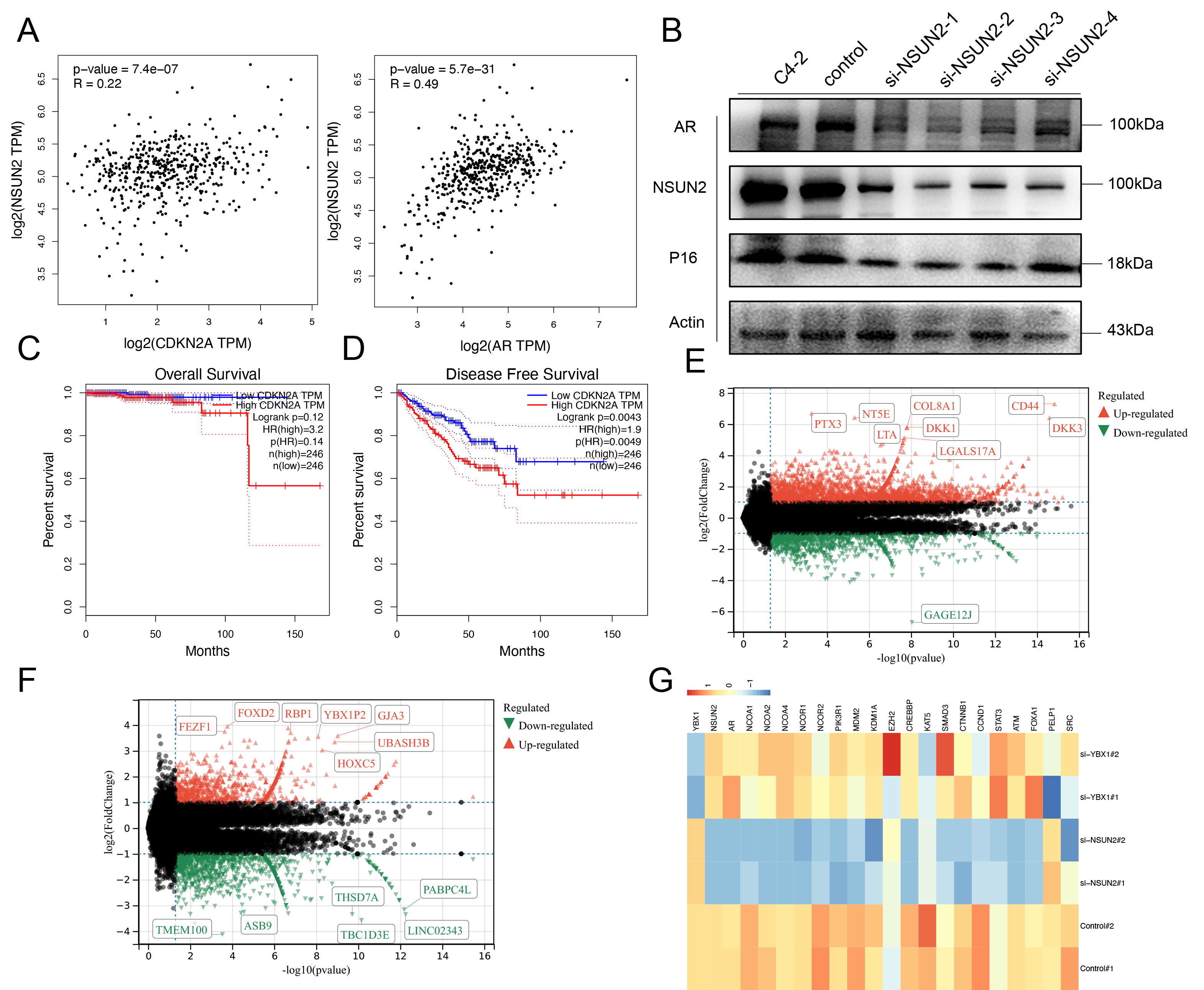

Supplement: Supplementary file 10 — Supplementary Figure 7 The action of NSUN2 on p16 does not affect its promotion of prostate cancer. [file CTM2-12-e1028-s008.tif]
