# Supplementary material for: Positive epigenetic regulation loop between AR and NSUN2 promotes prostate cancer progression
Source: Clin Transl Med. 2022 Sep 28;12(9):e1028. doi: 10.1002/ctm2.1028 (PMC9516604; doi:10.1002/ctm2.1028)
Supplement: Supplementary file 11 — Supporting Information [file CTM2-12-e1028-s001.docx]

**Antibodies:**

| Antibody | Cat No. | Concentration |
| --- | --- | --- |
| NSUN2 | 20854-1-AP | 1:500 |
| YBX1 | 20339-1-AP | 1:500 |
| Actin | Ab8227 | 1:1000 |
| m^5^C | Ab214727 | 0.5 - 2 µg/ml. |
| AR | Ab108341 | 1:500 |
| AR-V7 | 31-1109-00 | 1:1000 |
| FITC | F-2765 | 2 µg/mL |
| mCherry | M11241 | 2 µg/mL |
| HRP | SA00001-2 | 1:5000 |

**primers：**

**qPCR Primers:**

NSUN2-F：GAACTTGCCTGGCACACAAAT

NSUN2-R：TGCTAACAGCTTCTTGACGACTA

β-actin-F：CATGTACGTTGCTATCCAGGC

β-actin-R：CTCCTTAATGTCACGCACGAT

AR-F：CCAGGGACCATGTTTTGCC

AR-R：CGAAGACGACAAGATGGACAA

KLK3-F: TTTCCAATGACGTGTGTGCG

KLK3-R: ATCAGAATGACCCACGAGCAG

FOLH1-F: ACTCTCATCTGGCATTATCAGGG

FOLH1-R: GGCCTCTGGCTTGGGATAAT

AR intron1-F: GGGCCCAGGATTTTATCATCTCAGA

AR intron1-R: CACTGCCTAAATTGCCTGTACAAACA

AR intron2-F: TTTACATCTGCCCAATGCCAGG

AR intron2-R: GCTACAGTTGCAGGCACTCAGAAATA

AR-intron3-F GTCACTGAGCTGAAGGTAGTAGCT

AR-intron3-R GGGAAAGTCGGACTGTAGTCTCTC

AR intron4-F: GGATGCTCTTCTATATTTGCACACACAG

AR intron4-R: CAACCTCATGTTCTTTCCACCATACC

AR intron5-F: GACCTTGGTACTGCCATTTTCCTTCC

AR intron5-R: GTTAAAGGTCTTTCCCAAAGATGCTGAC

AR intron6-F: GGCCTTTTTGACTGGTTACAGCAG

AR intron6-R: CTCCTGTTGGCAGAAGAGGAAATCT

AR intron7-F: TTCTCTAGAGTCTGGCACCACCTG

AR intron7-R: GGACACTGTGACCCGTGTTCTTTT

**ChIP Primers:**

NSUN2-ChIP-F：GCGTACTGGTAGAGCGGC

NSUN2-ChIP-R：TCTTCGCGCGCAATCGT

**Probe synthesis Primers:**

AR probe-F：

TAATACGACTCACTATAGGGAGACTTTCCAGAATCTGTTCCAGAGCG

AR probe-R：GGCAGCCCCTTGCTGG

**RIP Primers:**

m^5^C RIP-PCR-F：CTTTCCAGAATCTGTTCCAGAGCG

m^5^C RIP-PCR-R：CCTGGGGCTAGTCTCTTGCT

**si-RNA:**

siNSUN2-1: 5’-UAAGAAAGAUGGCGUGUGU-3’

siNSUN2-2: 5’-GAGUUGGUAUCCUGAAGAA-3’

siNSUN2-3: 5’-GAGCGAUGCCUUAGGAUAU-3’

siNSUN2-4: 5’-GGAGACGGCACUAUGAGAA-3’

siYBX1-1: 5’-GGAACGGAUAUGGUUUCAU-3’

siYBX1-2: 5’-GCAGACCGUAACCAUUAUA-3’
